# Biochemical and Computational Assessment of Acute Phase Proteins in Dairy Cows Affected with Subclinical Mastitis

**DOI:** 10.3390/cimb45070338

**Published:** 2023-06-26

**Authors:** Aarif Ali, Muneeb U. Rehman, Saima Mushtaq, Sheikh Bilal Ahmad, Altaf Khan, Anik Karan, Amir Bashir Wani, Showkat Ahmad Ganie, Manzoor Ur Rahman Mir

**Affiliations:** 1 Department of Clinical Biochemistry, School of Biological Sciences, University of Kashmir, Srinagar 190006, India; buttaarif31@gmail.com; 2 Division of Veterinary Biochemistry, Faculty of Veterinary Sciences & Animal Husbandry, SKUAST-Kashmir, Alusteng, Shuhama 190006, India; sbilal07@gmail.com; 3 Department of Clinical Pharmacy, College of Pharmacy, King Saud University, Riyadh 11451, Saudi Arabia; muneebjh@gmail.com; 4 Veterinary Microbiology Department, Indian Veterinary Research Institute (IVRI), Bareilly 243122, India; saimushtaq88@gmail.com; 5 Department of Pharmacology & Toxicology, College of Pharmacy, King Saud University, Riyadh 11451, Saudi Arabia; altkhan@ksu.edu.sa; 6 Department of Surgery-Transplant and Mary & Dick Holland Regenerative Medicine Program, College of Medicine, University of Nebraska Medical Center, Omaha, NE 68198, USA; anik1432@gmail.com; 7 Division of Plant Biotechnology, SKUAST-Kashmir, Shalimar, Srinagar 190006, India; waniamir1647@skuastkashmir.ac.in

**Keywords:** subclinical mastitis, somatic cell count, acute phase proteins, principal component analysis, computational approaches

## Abstract

Subclinical mastitis (SCM) is a predominant form of mastitis wherein major visible signs of disease are absent. The present study aimed to determine acute phase proteins (APPs) like ferritin, C-reactive protein (CRP), and microalbumin (Malb) in 135 composite milk and serum samples of healthy (*n* = 25) and SCM (*n* = 110) cows. As bovine mastitis is an inflammatory disease, the present study also aimed at finding novel anti-inflammatory compounds from natural sources by repurposing approach using computational studies. The findings of the present study revealed substantial elevation (*p* < 0.001) in milk SCC and an increase in ferritin, CRP, and Malb (*p* < 0.001) in milk and sera of the SCM group as compared to healthy animals. Receiver operating characteristics of milk SCC, milk, and serum APPs unraveled statistically substantial alteration (*p* < 0.001). Further, SCC was correlated with milk APPs ferritin (r = 0.26 **, *p* < 0.002), CRP (r = 0.19 *, *p* < 0.02), and Malb (r = 0.21 *, *p* < 0.01). Additionally, milk SCC was correlated with serum ferritin (r = 0.28 **, *p* < 0.001), CRP (r = 0.16, *p* > 0.05), and Malb (r = 0.16, *p* > 0.05). The findings of molecular docking revealed that Chaetoglobosin U was the most effective molecule that showed the highest binding affinity (kcal/mol) of −10.1 and −8.5 against ferritin and albumin. The present study concluded that the estimation of cow-side tests, SCC, and APPs in milk/serum is suitable to detect SCM and screening herd community. Furthermore, Chaetoglobosin U could be developed as a promising anti-inflammatory inhibitor; however, further studies are required to validate these findings.

## 1. Introduction

Bovine mastitis is the persistent inflammatory state of the parenchyma of the mammary gland, usually accompanied by changes in the physicochemical properties of milk and pathological changes in the glandular tissue [1]. Subclinical mastitis (SCM) is the major form of mastitis and is bereft of any obvious manifestations. SCM is 15–40 times more predominant when compared with clinical mastitis, with infected cows becoming a major source of infection for healthier cows [2,3]. SCM causes huge economic losses as it decreases the quantity and quality of milk and makes it more challenging to identify infected animals from the herd [4]. Generally, SCM is detected by cow-side tests such as the California mastitis test (CMT), pH, electrical conductivity (EC), and somatic cell count (SCC) [5,6,7]. In dairy cows, SCC is considered the gold standard test to detect SCM and this test measures somatic cells present in milk, which are released mainly due to microbial invasion [8]. Somatic cells are typically white blood cells and mainly consist of 75% leukocytes, i.e., neutrophils, macrophages, lymphocytes, erythrocytes, and 25% epithelial cells [9]. During mastitis, these cells enter the mammary gland and the measurement of these cells acts as a reliable indicator for determining SCM [9].

Therefore, for the early diagnosis of mastitis, it is important to identify specific mastitis biomarkers, so that therapeutic intervention can be provided at an earlier stage to reduce disease severity as well as huge economic losses to the dairy industry. Following the tissue damage, a couple of systemic events happen, especially acute phase response (APR) that is initiated by macrophages or by blood monocytes of the damaged tissue, which secrete various mediators primarily cytokines and other pro-inflammatory molecules like interleukin-1 (IL-1), interleukin-6 (IL-6), and tumor necrosis factor-alpha (TNF-α) [10]. The utmost prominent variations that are associated with APR are the metabolic changes that increase the synthesis of a group of plasma proteins commonly known as acute phase proteins (APPs), largely synthesized by hepatocytes of the liver [11]. APP concentration changes due to internal or external encounters that include any kind of infection, swelling or inflammation, and any trauma or stress associated [12]. In dairy herds, the blood–milk barrier permeability is altered during the dry-off and oestrus phases, leading often to the seepage of blood proteins directly into the milk [8]. Consequently, the existence of APPs in milk recommends that there is a self-regulating relationship existing between SCC and APP [8].

Bovine ferritin is a positive APP primarily synthesized and secreted by hepatocytes. Serum ferritin in the body acts as an early sensitive marker for iron deficiency, as it senses the body’s iron stores [13]. In inflammation or infection, ferritin makes iron available to the cells to protect lipids, proteins, and DNA from probable iron toxicity [14]. Another APP is the C-reactive protein (CRP), produced by the liver and plays a vital role in phagocytosis [15]. The concentration of serum CRP increases during the early stages of infection [16]. On the other hand, to our best knowledge, no study has reported the use of Malb as a milk or serum marker of SCM in dairy cows.

Although of the recent advances made in diagnostic procedures, bovine mastitis remains a major problem in dairy herds, despite the widespread implementation of programs to control mastitis. However, a major hurdle to controlling mastitis has been caused due to injudicious and long-term antibiotic use with several antibiotic-resistant bacteria have emerged therefore leading to treatment failure in dairy cattle. Therefore, it is important to identify, alternate compounds from natural sources to tackle this inflammatory disease. In such a scenario, computational approaches have significantly established the rationale for identifying compounds that can be repurposed as novel inhibitors targeting proteins associated with anti-inflammatory diseases.

The present study aimed to evaluate APPs in milk and serum of cross-bred Holstein-Friesian (HF) animals affected with SCM, which could act as viable markers for the diagnosis of SCM at an early stage. The study also emphasized finding the association of SCC with APPs based on data from cows in a dairy herd. Thus, rapid tests for the estimation of APPs can prove as sensitive diagnostic tools to identify SCM and to monitor/improve herd health which could save the dairy industry from huge economic losses and increase farm revenue.

Similarly, the present study attempted to identify novel bioactives that can be repurposed to treat this inflammatory disease, and, hence, many fungal compounds were selected. In this study, absorption, distribution, metabolism, excretion, and toxicity (ADMET) analysis of fungal bioactives was determined. Furthermore, the present study determined the binding affinity (kcal/mol) of bovine ferritin and albumin docked against various fungal bioactives. In addition, the topographic and molecular dynamic behavior of the proteins was evaluated by bioinformatic methods. Moreover, the computational approaches allow the identification of effective drug targets and pave the way for discovering potential drugs.

## 2. Materials and Methods

### 2.1. Sampling Method and Sample Size

A cross-sectional study was conducted in the villages surrounding Ganderbal district (Jammu and Kashmir, India) to determine biomarkers in milk and serum for early screening of bovine SCM.

A simple random sampling technique was used to collect samples from the study area and a total of 135 lactating Holstein-Friesian crossbred dairy animals were taken for this study. Milk and blood samples were taken from HF dairy cows and collected as per the protocol suggested [17]. Before collection of samples, teats were properly washed with a moistened cotton pad soaked with 70% ethyl alcohol. Initial three to four squirts of milk were taken out from each quarter to reduce chances of bacterial contamination and a total of 15 mL fresh milk was aseptically drawn from quarters. The composite milk samples (CMS) were immediately examined for color and further subjected to screening tests, viz., CMT, pH, and EC. 

The remaining 5 mL of milk was aseptically taken in a sterile falcon tube for estimation of somatic cell count (SCC) and acute phase proteins (APPs). Similarly, 5 mL of blood drawn from the jugular vein of each animal was collected in clot activator tubes (RAPID TM) for serum extraction. The milk and blood samples were transported to the Division of Veterinary Biochemistry SKUAST-K, Shuhama Alusteng, J&K in a cooler packed with ice packs. Blood in clot activator tubes was subjected to centrifugation for 10 min at 3000 rpm for harvesting serum, which was then transferred into a 1.5 mL microcentrifuge tube. The milk samples and serum samples were stored at −80 °C immediately for further use. The overall distribution of composite milk samples (CMS) collected from selected dairy cows and put forth for various analyses is shown in Table 1. The Institutional Animal Ethical Committee (IAEC) approved all pertinent animal health procedures in this study, according to the relevant laws and institutional regulations (No: DST/SSTP/12th plan/150/J&K date: 27 January 2016).

### 2.2. Screening Tests

#### 2.2.1. California Mastitis Test (CMT)

The crossbred dairy cows were examined for mastitis based on clinical examination and the California mastitis test (CMT). CMT was performed based on the protocol described by Ali et al. [7]. CMT is a simple, quick, and easy-to-perform cow-side diagnostic test to detect SCM. This test correctly predicts the somatic cell count in the milk of dairy animals. Approximately 3 mL of composite samples of milk were obtained from each quarter and mixed in a CMT paddle after a few initial squirts of foremilk were discarded. After this, an equivalent of CMT reagent (3% sodium dodecyl sulfate) was put into the paddle that was rotated gently in a horizontal plane for about 10–30 s to mix the contents. The reagent operates by disrupting the cell membrane of cells allowing the DNA in those cells to react with the test reagent, forming a gel. Based on gel formation, the results were recorded as negative (no gel formation) and positive (gel formation) with scores ranging from 1 (weak positive), 2 (distinct positive), and 3 (strong positive). The severity of infection is determined by the intensity of the formed gel.

#### 2.2.2. pH

A portable digital pH meter was used to measure the pH of milk samples (Eutech, Singapore). A milk pH of more than 6.7 was used as a cut-off point to distinguish subclinical infected animals from healthy [1].

#### 2.2.3. Electrical Conductivity (EC)

The milk conductivity was measured using a portable digital EC meter (Eutech, Singapore). Milk conductivity greater than 4.44 mS/cm was used to distinguish between healthy and SCM dairy cows [1].

#### 2.2.4. Somatic Cell Count (SCC)

The gold standard diagnostic test for identifying subclinical mastitis is the estimation of somatic cell count in milk. The reference method that has been utilized in this work to detect SCC in milk was by DeLaval cell counter (DCC; DeLaval International AB, Tumba, Sweden). The detection of SCC in bovine milk at 4 °C and 37 °C has been well validated by the DCC method. The DeLaval cell counter is a portable automated cell counter which measures somatic cells (leukocytes) within a range of 10,000–4,000,000 cells/mL. About 60 µL of the milk sample was loaded into the DeLaval cell counter^®^ (DCC) cassettes which cross through various portions of the cassette and ultimately utilizes (10 µL) for analysis. The cassette was then loaded into the digital somatic cell counter and the “start” button was pressed to obtain the number of somatic cells (cells/µL) present in composite milk samples. After a delay of 45 s, the result was displayed on the screen, which showed cell numbers per microliter (µL) of milk. The results were then multiplied by a factor of 1000 to obtain milk SCC in units of cells/mL. An SCC of >200,000 was used as a cut-off point for differentiating subclinical infected animals from healthy animals [1].

#### 2.2.5. Acute Phase Proteins

The concentration of APPs (Ferritin, CRP, and Malb) in milk and serum was determined according to the instructions of the manufacturer. Immunoassay-based fluorescence kits (QAYEE-BIO, Life Science, Co., Ltd., Daegu, South Korea) were used for the detection of APPs in the present study.

### 2.3. Statistical Analysis

Descriptive statistical analysis of milk SCC and APPs have been performed through GraphPad Prism (version 8) software. A non-parametric test (Mann–Whitney) of significance (with significance accepted at *p* < 0.05) was measured by GraphPad (version 8) software. Multivariate, univariate, and biomarker analysis were performed through MetaboAnalyst software (www.metaboanalyst.ca; accessed on 5 December 2022) an online tool was used to estimate principal component analysis (PCA), sparse partial least discriminant analysis (sPLS–DA), and orthogonal partial least square discriminant analysis (oPLS–DA). In these procedures, data were normalized or filtered, and a 95% confidence interval was used. The receiver operating characteristics (ROC) analysis was used to determine the test’s sensitivity and specificity. A VIP (variable importance in the projection) plot was used in the PLS–DA model to rank the metabolites based on their importance in distinguishing the SCM group from the healthy group of cows.

## 3. Computational Approaches

### 3.1. Ligand Selection

In the present study, various fungal bioactives (Asperflavin, Asperlin, Austinolide, Cordyol E, Khusinol B, Luteoride E, Cytochalasin E, Chaetoglobosin U) were selected.

Two control drugs Penicillin G and Doxycycline were also considered in this study. The 2D structure of all the compounds was retrieved from PubChem (http://pubchem.ncbi.nlm.nih.gov/compound; accessed on 5 December 2022) in simple data format (SDF).

### 3.2. Drug Likeliness

In this study, the SwissADME tool (http://swissadme.ch/index.php; accessed on 5 December 2022) was used to evaluate the drug-likeliness of fungal bioactives. Lipinski’s rule of five (RO5) was used to predict hits based on different filtering parameters [18,19]. The RO5 includes five attributes, i.e., molecular weight (<500 g/mol, *A log P* < 5, and the number of hydrogen bond acceptors and donors should be less than 10 and 5).

### 3.3. pkCSM

The determination of ADMET properties is crucial in the discovery of lead drug molecules. In the current study, the ADMET properties of molecules were assessed using the pkCSM server (https://biosig.lab.ug.edu.au/pkcsm; accessed on 5 December 2022).

## 4. Molecular Docking

### 4.1. AutoDock Vina

In the present study, Auto Dock Vina (UCSF-Chimera, version 4.2.6) was used for molecular docking analysis. A standard protocol was used for the docking analysis of protein molecules with drugs and phytocompounds as mentioned [20]. The protein structures were retrieved from the protein data bank with PDB IDs of 7U5L (ferritin) and 3V03 (albumin). In the protein data bank, no PDB ID was available for bovine CRP. In the present study, proteins were prepared by dehydrating them, then adding polar hydrogen and Gasteiger charges. Similarly, Gasteiger charges and torsion were added to the compounds selected as ligands in the input option. The ligand and proteins were both saved in the pdbqt format. During docking analysis, the protein was chosen as a macromolecule and phytocompounds as its ligands. The center dimensions in the current study for ferritin (x = 101.149, y = 127.221, z = 162.022) and albumin (x = 64.296, y = 25.805, z = 32.089) with spacing of 0.387, respectively.

### 4.2. Computed Atlas of Surface Topography of Proteins (CASTp)

CASTp identifies and reveals the complete topographic properties of a protein molecule (http://sts.bioe.uic.edu; accessed 21 July 2022). CASTp employs the alpha shape method with a default radius of 1.4 A° and requires PDB format or a 4-letter PDB ID of the protein as input.

### 4.3. iMODS

The iMODS server (http://imods.chaconlab.org; accessed on 5 December 2022) evaluated the molecular simulation in normal mode. This server assists us in evaluating different conformational attributes such as eigenvalues, deformity, B factor, variance%, and co-variance map.

## 5. Results

In this study, CMT, pH, and EC were used as confirmatory tests to detect SCM in dairy animals. In the present study, CMT was found to be negative in the healthy group, and animals were given a CMT score of (−) or (0), but animals with SCM were given a CMT score of weak positive (+), distinct positive (++), and strong positive (+++). Positive quarters were those that presented with a CMT score of (+) or higher.

In the selected animals, the values of pH, EC, and SCC in milk greater than 6.7, 4.44 mS/cm, and 200,000 cells/mL were used as an optimal cut-off point for differentiating healthy from SCM-infected animals. The dairy animals that showed values pH, EC, and SCC in milk higher than the optimal point were grouped under the SCM group.

### 5.1. Somatic Cell Count

**Figure 1:**
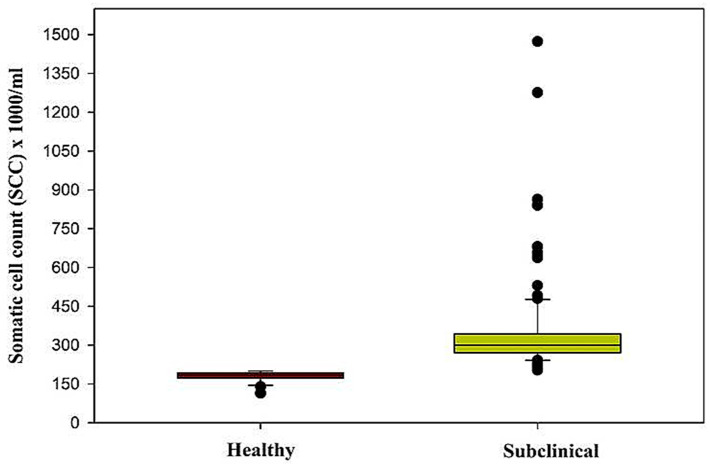
Box plot of somatic cell count in healthy and subclinical mastitis animals.

The measurement of SCC in milk is considered a gold standard test for detecting SCM (Table 2). An SCC value of >200,000 somatic cells/mL of milk was put as a cut-off point to separate the healthy group from the SCM group. In the present study, SCC in the healthy group ranged from 114–200 cells/mL with a median of 184 cells/mL and a mean value of 180.2 ± 4.12 cells/mL, while as in SCM, the concentration ranged from 203–1473 cells/mL with a median of 300 cells/mL and a mean value of 348.3 ± 17.27 cells/mL, respectively. Substantial elevation (*p* < 0.001) in SCC concentration was observed in SCM dairy cows as compared to the healthy group (Table 3 and Figure 1).

### 5.2. Acute Phase Proteins

In this study, the profile of APPs (Ferritin, CRP, and Malb) were studied to determine their concentrations in milk and serum of healthy and SCM-affected Holstein-Friesian cows for early diagnosis.

### 5.3. Milk APPs

The milk samples of both groups were used for quantitative estimation of ferritin, CRP, and Malb. In the present study, the skewness statistics showed the higher distribution of ferritin and CRP in the milk of the SCM group as compared to the healthy group as scores clustered to the right. Furthermore, skewness statistics of Malb revealed higher distribution in the SCM group as scores gathered to the right as compared to the healthy group who had a minor range of scores having negative skewness value.

**Figure 2:**
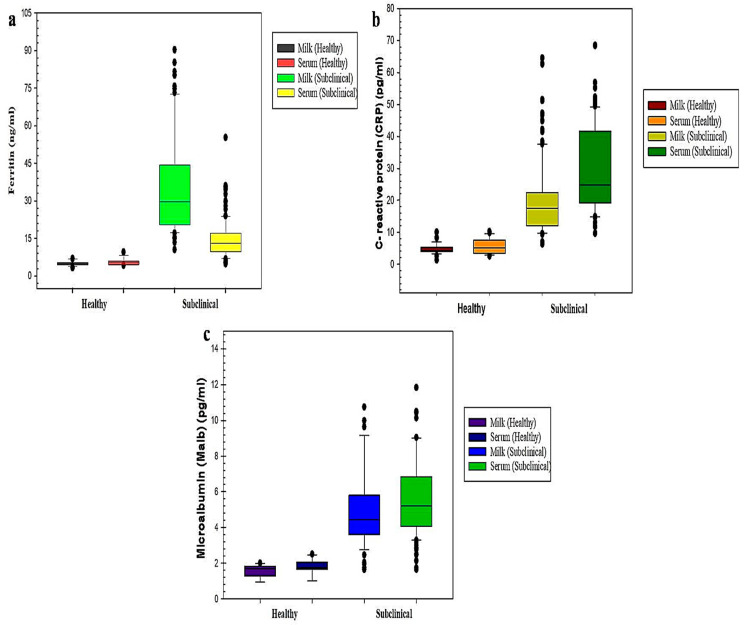
Box plot of acute phase proteins in milk and serum of healthy and subclinical infected animals. The box plots represent the concentration of ferritin (**a**), CRP (**b**), Malb (**c**) in milk and serum of dairy cows.

Further, the concentration of ferritin in the milk of healthy animals had a mean value of 5.07 ± 0.19 ng/mL, whereas in SCM the mean concentration was 35.10 ± 1.89 ng/mL, respectively (Table 4 and Figure 2a–c).

Similarly, CRP concentration in the milk of healthy had a mean value of 4.64 ± 0.34 pg/mL, whereas in SCM it was 20.19 ± 1.14 pg/mL, respectively (Table 4 and Figure 2a–c).

Moreover, the concentration of Malb in the milk of healthy animals had a mean value of 1.59 ± 0.06 pg/mL, whereas in SCM the mean concentration was 5.03 ± 0.21 pg/mL, respectively (Table 4 and Figure 2a–c).

Substantial elevation (*p* < 0.001) in milk APPs (Ferritin, CRP, Malb) was observed in SCM dairy cows as compared to the healthy animals. Descriptive statistics of milk APPs concentration in healthy and SCM-affected Holstein-Friesian dairy cows are presented in Table 4 and Figure 2a–c.

### 5.4. Serum APP’s

In this study, the skewness statistics showed the higher distribution of serum ferritin in SCM as compared to the healthy group, as scores clustered to the right. Further, skewness statistics of CRP in the SCM group were found to be lower in comparison to the healthy group which showed a slight increase in value. Furthermore, skewness statistics of Malb revealed higher distribution in the SCM group as scores clustered to the right, whereas the lower range of scores with negative skewness values was observed in the healthy group.

The concentration of ferritin in the serum of healthy animals ranged had a mean concentration of 5.67 ± 0.28 ng/mL, whereas in SCM the mean concentration was 14.54 ± 0.77 ng/mL, respectively. Similarly, CRP in the serum of healthy animals had a mean concentration of 5.42 ± 0.47 pg/mL, whereas in SCM the mean value was 29.71 ± 1.25 pg/mL, respectively (Table 5 and Figure 2a–c).

Moreover, the concentration of Malb in the serum of healthy animals had a mean value of 1.77 ± 0.08 pg/mL, whereas in SCM the mean concentration was 5.61 ± 0.21 pg/mL, respectively.

Substantial elevation (*p* < 0.001) in ferritin, CRP, and Malb concentration in serum was observed in the SCM group in comparison to the healthy group (Table 5 and Figure 2a–c).

### 5.5. Receiver Operating Characteristics (ROC) Analysis

ROC curves are the overview of the set of possible groupings of sensitivity and specificity likely for predictors [21]. The area under the curve (AUC) for SCC was 1 with a standard error of 0.00. The results prove that 100% of cases could be forecasted as mastitis with SCC being a marker (*p* < 0.001). At a cut-off point of 202,000 cells/mL of milk, the sensitivity and specificity are 100%. However, at cut-off scores of 204,000, 211,000, 229,000, 233,000, 237,000, 240,000, and 242,000 cells/mL, the sensitivity found was 100%, whereas the respective specificity was 99.09%, 98.18%, 96.36%, 95.45%, 94.55%, 90%, and 90%, respectively. With the increase in the SCC of milk beyond this point, the specificity decreased.

The ROC analysis of milk APPs (ferritin, CRP, Malb) is at a cut-off value of 8.74, 8.82, and 2.03. The AUC for ferritin is 1 for CRP 0.99 and in the case of Malb, it is 0.98 which is considered to be good. The sensitivity and specificity of milk parameters for differentiating between mastitis and normal dairy cows was 100% for the ferritin test, while milk CRP showed 93% and 96%, and in the case of Malb, it was 96% and 100%, respectively.

**Figure 3:**
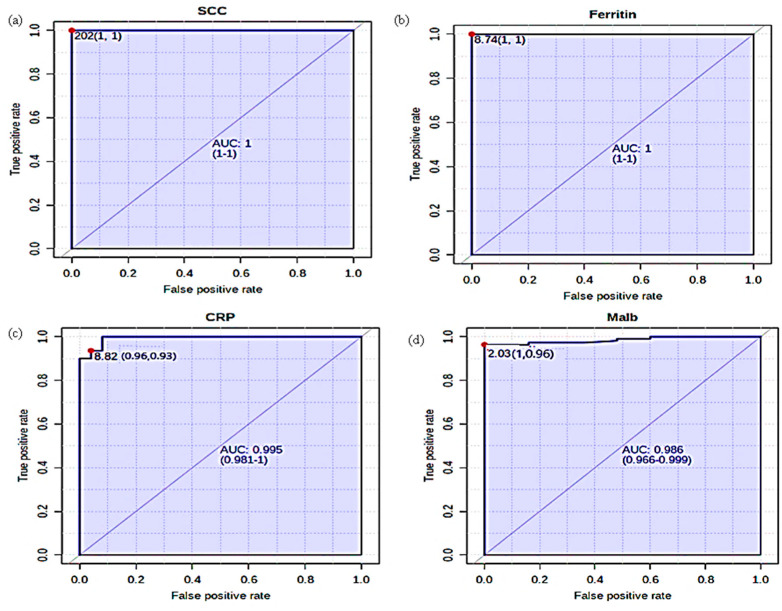
ROC curve analysis for SCC and acute phase proteins in milk.

**Figure 4:**
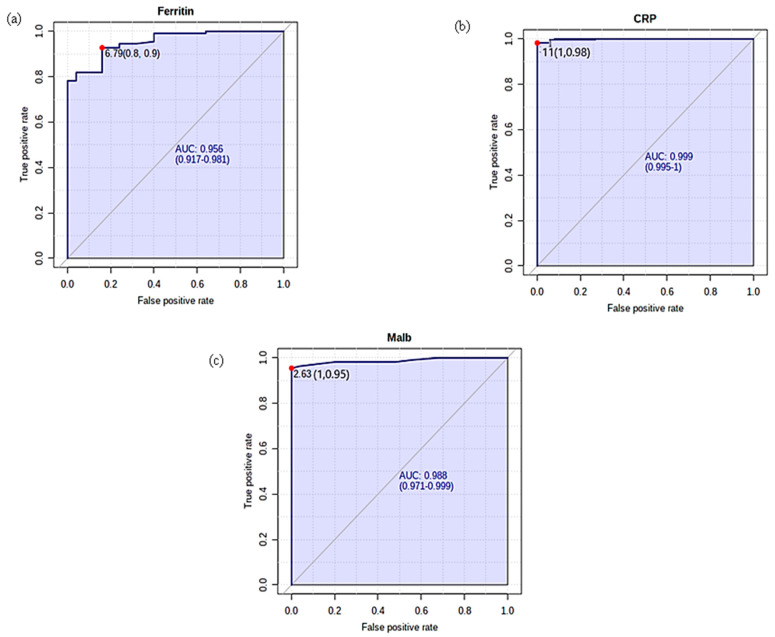
ROC curve analysis for serum acute phase proteins.

ROC analysis of serum APPs (ferritin, CRP, Malb) is at a cut-off value of 6.79, 11, and 2.63. The AUC for ferritin was 0.95 for CRP 0.99 and 0.98 for Malb, which determines the reliability of a very good test. The sensitivity and specificity of ferritin, CRP, and Malb in serum for differentiating between mastitis and normal cows were 92% and 84%, 98% and 100%, and 95% and 100%, respectively. ROC curve analysis for SCC and APPs in milk is shown in Table 6 and Figure 3a–d, whereas Table 6 and Figure 4a–c represent ROC analysis for sera APPs.

### 5.6. Association between SCC and APPs

To study the association of SCC with milk and serum APPs, Pearson’s correlation technique was used. Correlation matrix results showed low positive significant correlations of SCC with APPs: Ferritin (r = 0.26 **, *p* < 0.002), CRP (r = 0.19 *, *p* < 0.02), and Malb (r = 0.21 *, *p* < 0.01) concentrations in milk. The correlation matrix results revealed that a low positive noteworthy association (r = 0.28 **, *p* < 0.001) exists amid serum ferritin and milk SCC. However, no correlation of milk SCC was found with CRP (r = 0.16, *p* > 0.05) and Malb (r = 0.16, *p* > 0.05) of serum samples. The correlation coefficient between SCC, ferritin, CRP, and Malb in milk and serum of healthy and SCM dairy animals is presented in Table 7 and Table 8.

### 5.7. Principal Component Analysis (PCA)

**Figure 5:**
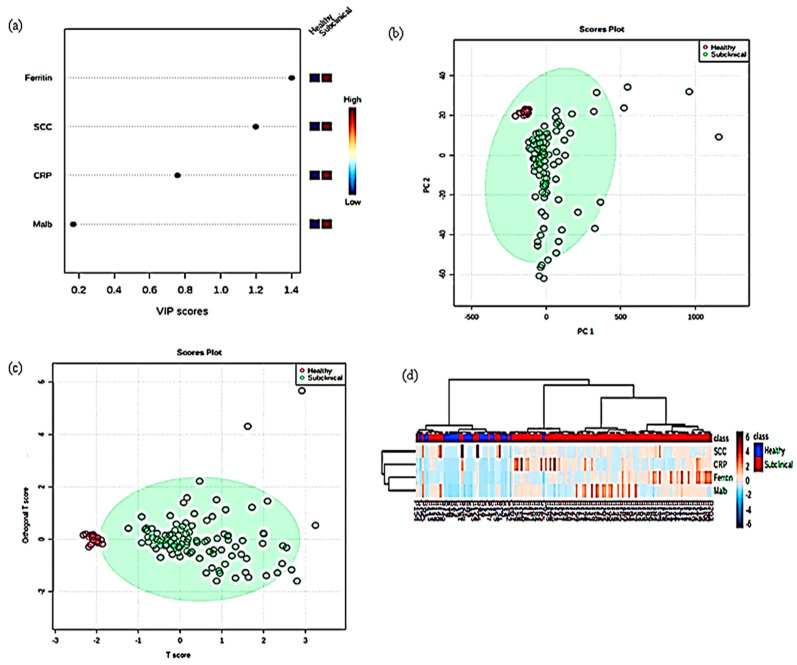
Principal component analysis of milk SCC and APPs. (**a**) VIP scores (**b**) sPLS–DA (**c**) oPLS–DA (**d**) Heat map.

**Figure 6:**
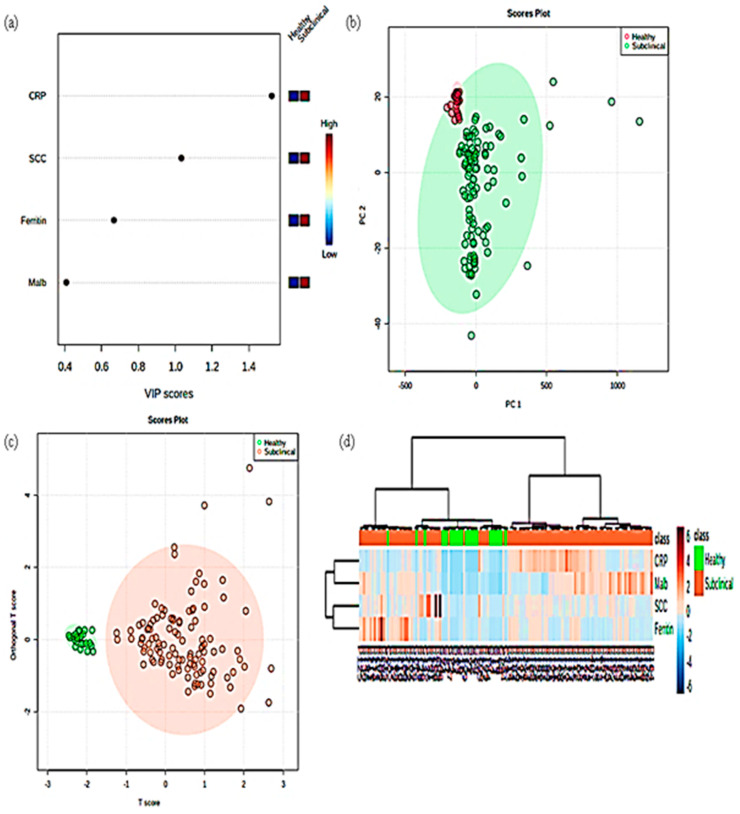
Principal component analysis of serum APPs. (**a**) VIP scores (**b**) sPLS–DA (**c**) oPLS–DA (**d**) Heat map.

In this study, by a combination of univariate and multivariate analysis, we compared the subclinical mastitic group with the healthy group. In this study, Figure 5a and Figure 6a represent VIP plots of metabolites in dairy cow milk from healthy and subclinical cows. The most powerful group discriminators are the metabolites with the highest VIP values. VIP values greater than 1 are considered significant, and VIP values greater than 2 are considered extremely significant. The VIP plots show the top four most important metabolites that distinguished the two groups. The greater the VIP value, the greater the contribution of metabolite molecules in differentiating subclinical mastitis cows from healthy animals. The VIP plots indicate that ferritin and somatic cell count in milk and sera CRP are the strongest discriminators for distinguishing mastitis animals from healthy.

We performed a supervised PCA, sparse version of the partial least-squares discriminant analysis (sPLS–DA) among the two groups to assess the performance classification of metabolite sets (Figure 5b and Figure 6b). The findings revealed that the main principal components (PC1 and PC2) explained 99.6% of the variability in milk samples and 99.8% of the variability in serum samples. Our study had 2 components, PC1 and PC2, which accounted for 98.2% and 1.4% variability in milk samples, whereas in serum the variability was 99.1% and 0.7%, respectively. The PLS–DA model’s classification performance was evaluated using the perf function and five-fold cross-validation repeated ten times, and the overall and balanced error rates per class were less than 0.2 within components. To evaluate differences among healthy and mastitis-infected animals, we used orthogonal partial least squares discriminant analysis on the milk and serum of healthy and subclinical infected animals (o–PLSDA). The results (shown in Figure 5c and Figure 6c) show the two groups’ similarities and separations. Moreover, the model evaluation revealed a high fitting accuracy (*p* < 0.001), with a 30.9% variation between the two groups, and separated pure milk from mastitis-infected milk (x-axis). Furthermore, the analysis revealed a 19.9% (milk) and 24.3% (serum) difference between groups, indicating a high level of variation in the detected metabolites and a good separation. In addition, we created a heat map to organize the accumulation levels of the top four contributors across the various spatial samples (Figure 5d and Figure 6d). Based on this visualization, many of these metabolites in both groups showed a decreasing trend from the outer to the inner layers. In general, the two groups showed similar trends in metabolite spatial distribution, though distinct patterns were observed in each group. The principal component analysis of milk and serum APPs is shown in Figure 5 and Figure 6.

### 5.8. Computational Analysis

#### 5.8.1. Drug Likeliness

In the present study, all the fungal bioactive compounds followed the parameters of drug likeliness. All the bioactive compounds followed Lipinski’s rule of five except Chaetoglobosin U showed one violation with a molecular weight greater than 500 g/mol. The drug-likeness properties of bioactive compounds and drugs are shown in Table 9.

#### 5.8.2. ADMET

In this study, all the bioactives showed good human intestinal absorption (HIA), as the scores were higher than 30% (poor absorption). The results of the blood–brain barrier (BBB) indicate that compounds with values higher than 0.3 can cross BBB, whereas values < −1 depict poor distribution to the brain. The bioactive compounds showed good findings for water solubility (*log* S). The epithelial colorectal adenocarcinoma cell line (Caco-2) describes the absorption of an oral drug entity, as values higher than 0.90 indicate higher permeability. Asperflavin, Cordyol E, Khusinol B, Luteoride E, and Chaetoglobosin U showed a favorable Caco-2 value in this study, in contrast to the other compounds’ negative results. Skin permeability (*log* Kp) is an important predictor in this study, and scores > −2.5 indicate lower skin permeability. Luteoride E, Chaetoglobosin U, and Penicillin G showed hepatotoxicity in this study, while the other compounds did not affect the liver. Similarly, while the remaining compounds reported inactive activity, Luteoride E, Asperlin, Cordyol E, Khusinol B, and Cytochalasin E were active for carcinogenicity. In the present study, Table 10 represents the ADMET properties of the bioactives and the drugs.

### 5.9. Molecular Docking

**Figure 7:**
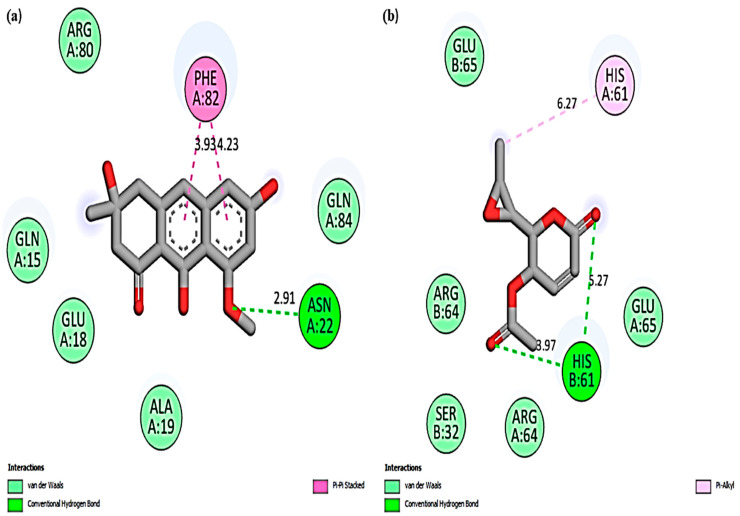

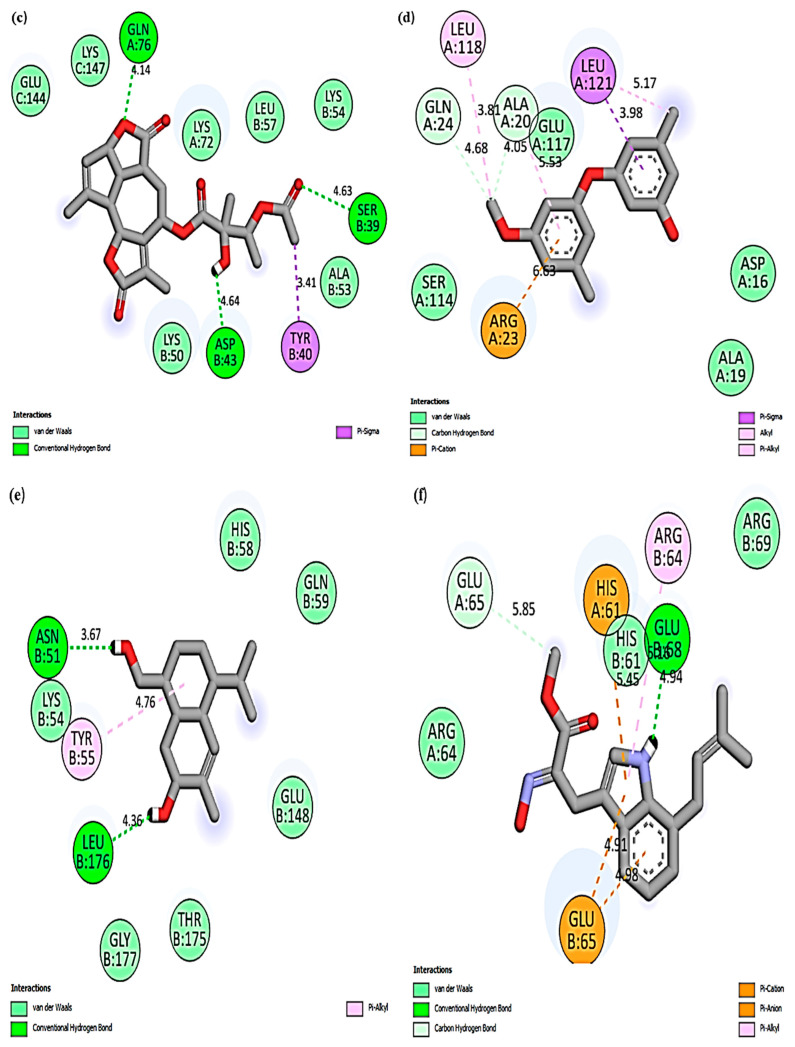

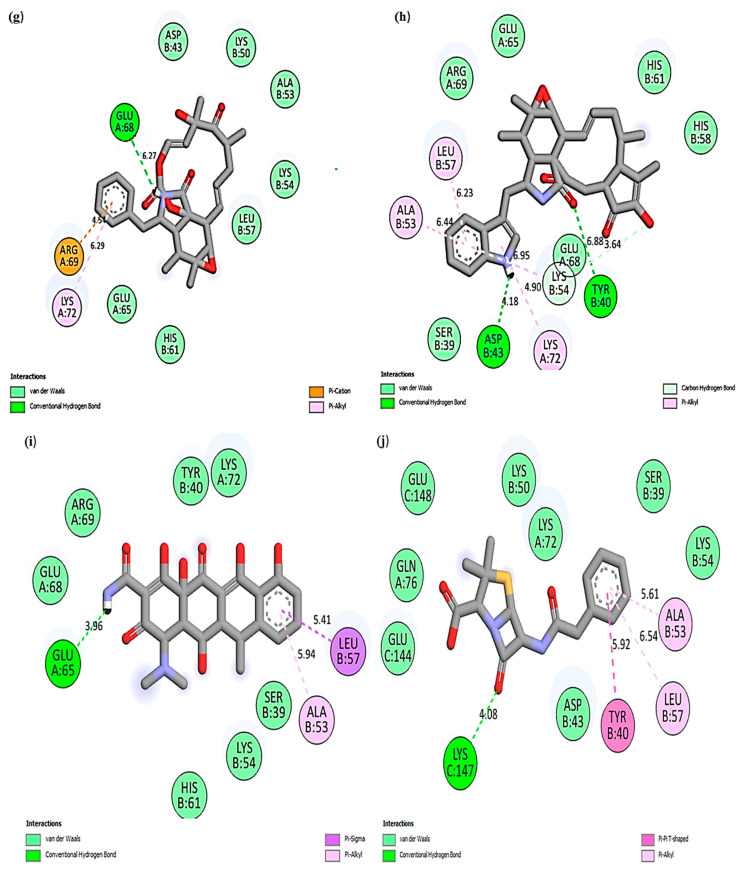
A 2D molecular interaction of bovine ferritin with (**a**) Asperflavin; (**b**) Asperlin; (**c**) Austinolide; (**d**) Cordyol E; (**e**) Khusinol B; (**f**) Luteoride E; (**g**) Cytochalasin E; (**h**) Chaetoglobosin U; (**i**) Penicillin G; (**j**) Doxycycline.

**Figure 8:**
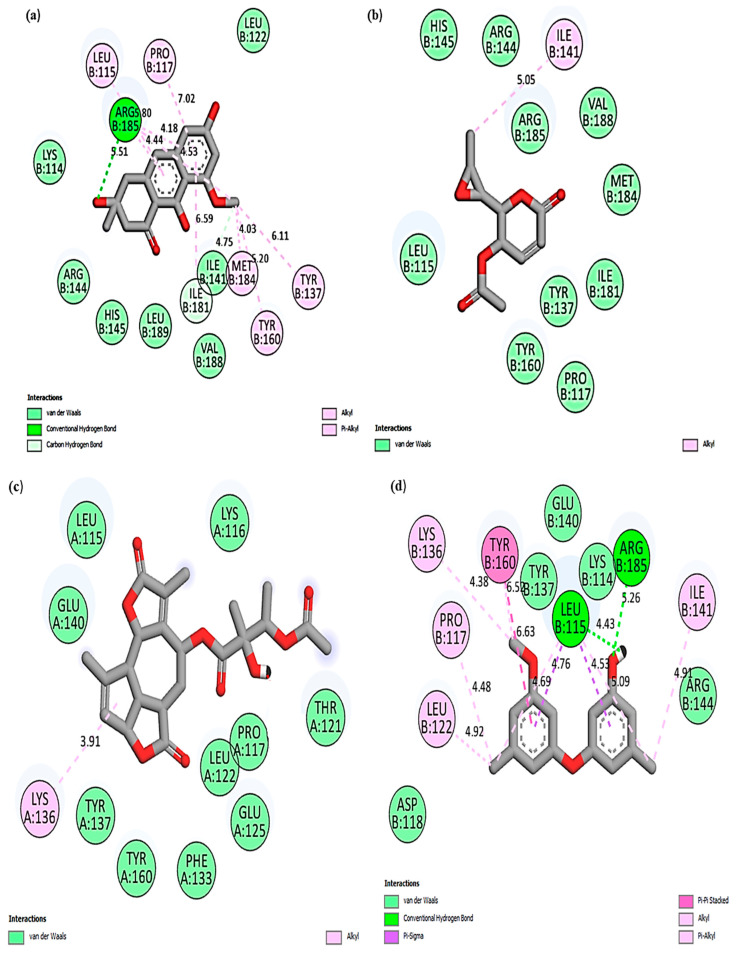

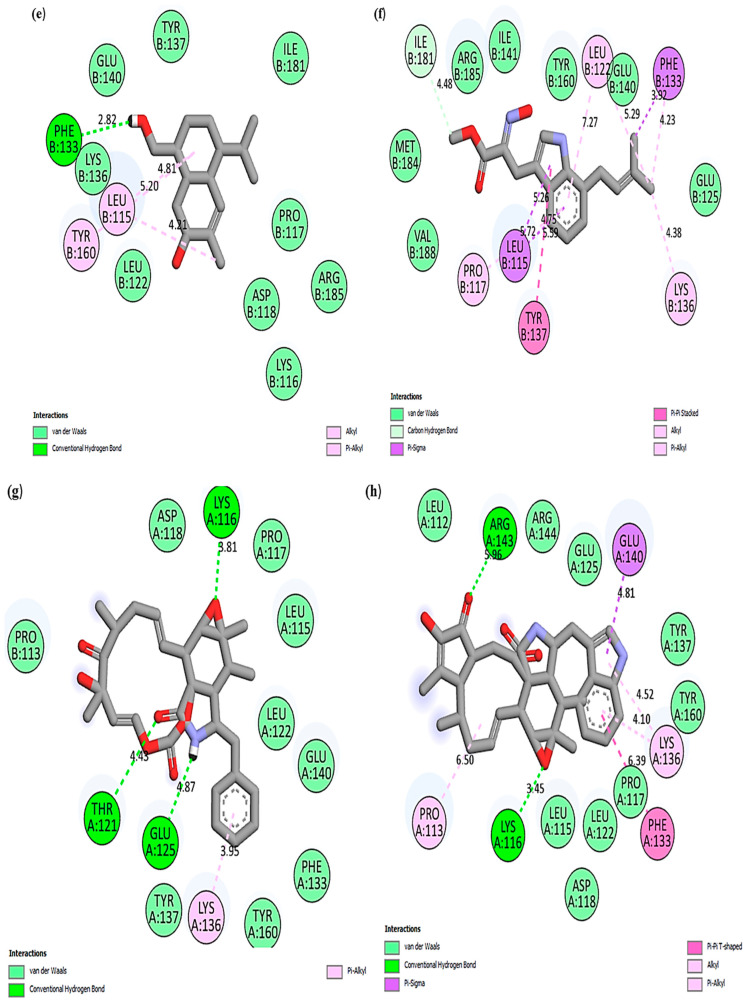

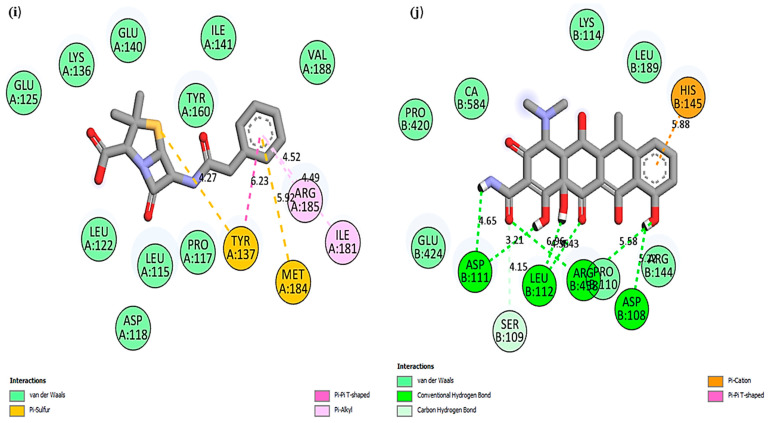
A 2D molecular interaction of bovine albumin with (**a**) Asperflavin; (**b**) Asperlin; (**c**) Austinolide; (**d**) Cordyol E; (**e**) Khusinol B; (**f**) Luteoride E; (**g**) Cytochalasin E; (**h**) Chaetoglobosin U; (**i**) Penicillin G; (**j**) Doxycycline.

In this study, Chaetoglobosin U was the most effective compound and showed the highest binding affinity (kcal/mol) of −10.1 and −8.5 when docked against bovine ferritin and albumin. Cytochalasin E was the second most effective compound with a binding affinity (kcal/mol) of −9.3 and −7.5 against ferritin and albumin. Among the bioactives, the least binding affinity was shown by Asperlin, with docking scores of −6.3 kcal/mol (ferritin) and −5.4 kcal/mol (albumin), respectively. Moreover, among the drugs, Doxycycline showed the highest binding affinity (kcal/mol) of −8.1 and −6.9 against ferritin and albumin, whereas Penicillin G reported the lowest with −7.9 (ferritin) and −6.4 (albumin), respectively. The binding affinities of all the bioactives docked against bovine ferritin and albumin are shown in Table 11. In the present study, Figure 7 and Figure 8 represent the 2D molecular interaction of fungal bioactives docked against bovine ferritin and albumin.

### 5.10. CASTp

**Figure 9:**
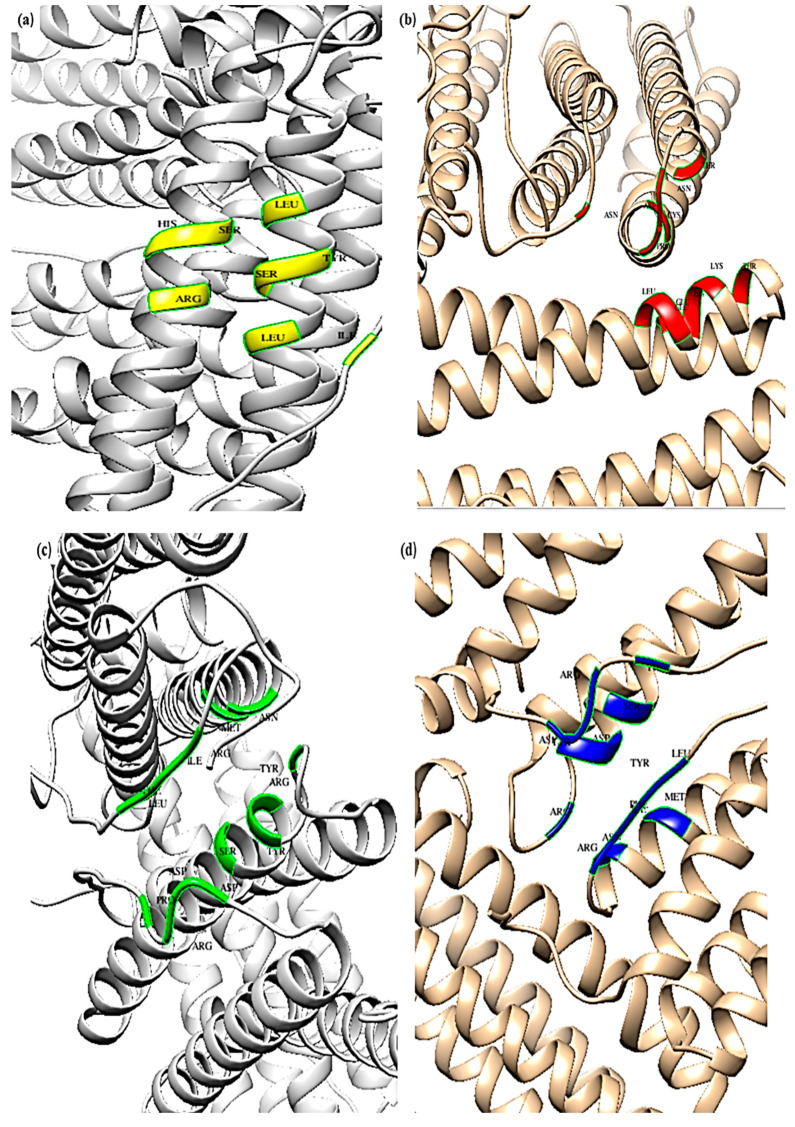

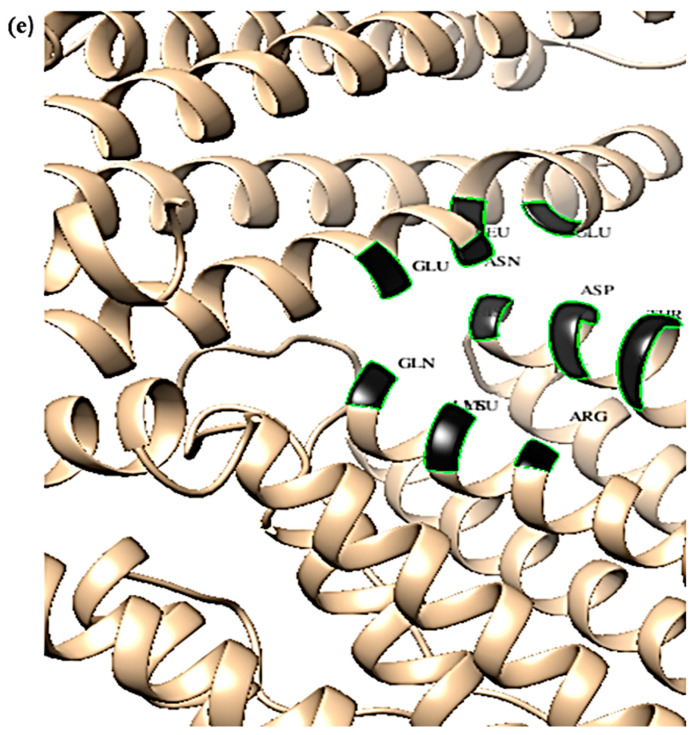
(**a**–**e**) CASTp data analysis of top five binding sites of ferritin.

**Figure 10:**
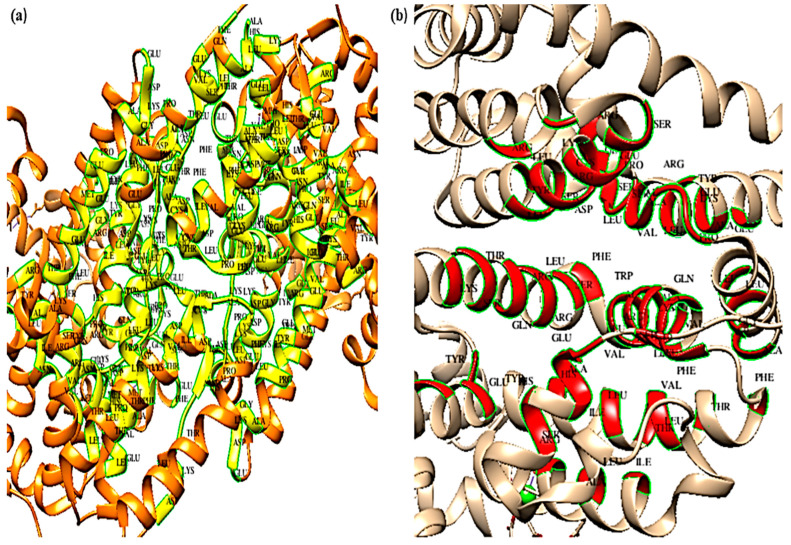

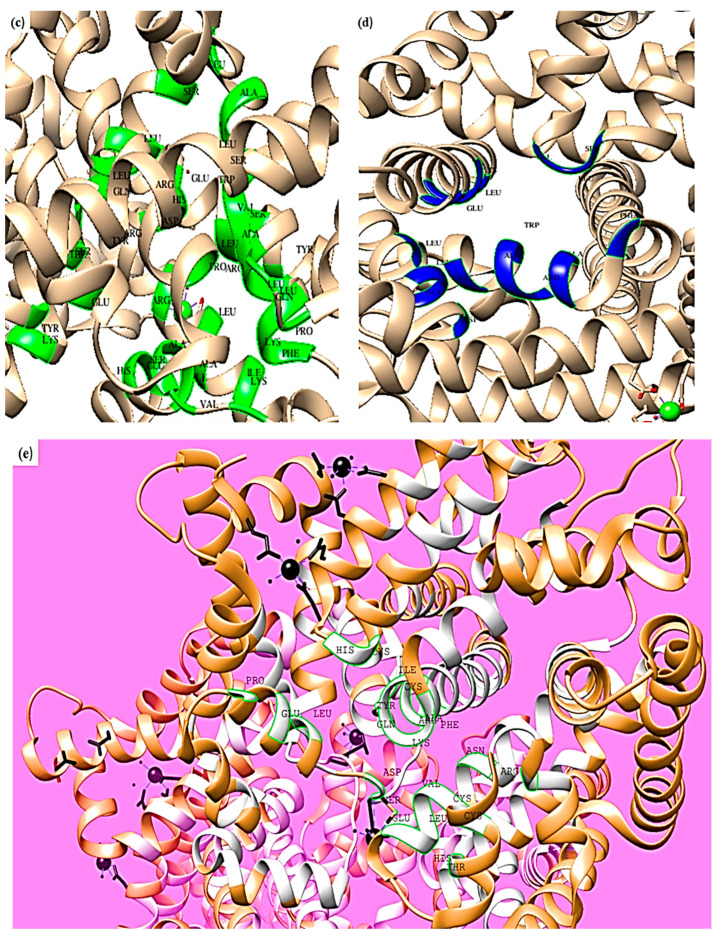
(**a**–**e**) CASTp data analysis of top five binding sites of albumin.

The top five binding pockets of the bovine ferritin and albumin proteins are shown in Figure 9 and Figure 10 along with their respective molecular surface (MS) volumes, pocket molecular surface (MS) areas, openings, and molecular surface (MS) mouth areas. Figure 9a–e and Figure 10a–e depict the topmost five binding sites of ferritin and albumin, respectively. The top five binding sites’ pocket imprints are displayed in different colors (yellow, red, green, blue, and black). The pocket panel contains significant residues, and drugs may be used to target these areas. The CASTp data is displayed in Table 12, along with information about the binding pockets’ volume, area, and openings.

### 5.11. iMODS

**Figure 11:**
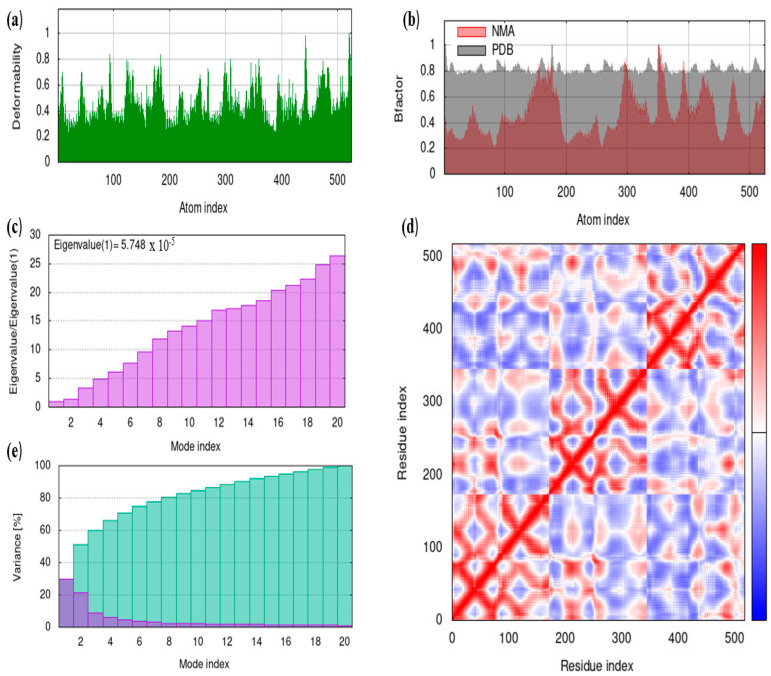
Molecular dynamics analysis of ferritin by iMODS. (**a**) Deformability (**b**) B-factor (**c**) Eigenvalue s (**d**) Covariance matrix (**e**) Variance.

**Figure 12:**
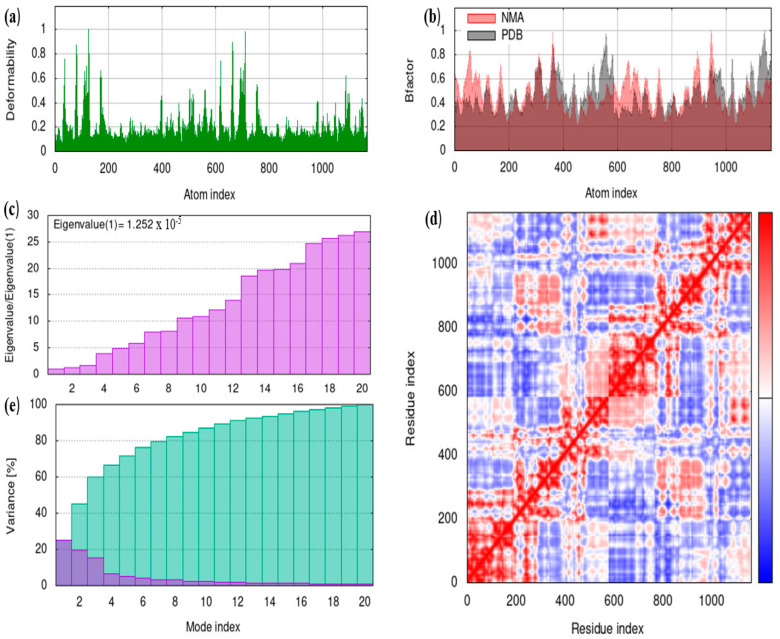
Molecular dynamics analysis of albumin by iMODS. (**a**) Deformability (**b**) B-factor (**c**) Eigenvalues (**d**) Covariance matrix (**e**) Variance.

In the present study, molecular dynamics of bovine ferritin and albumin was evaluated via the iMODS server and are illustrated in Figure 11 and Figure 12. The graph’s peaks correspond to regions of the protein that are deformable (main-chain deformity) (Figure 11a and Figure 12a). Since the hinges in the protein were not essential, the structure remained stable, and the regions with high deformability show where the chain hinges are. In this study, the B-factor evaluates the molecule’s ability to deform at each residue (Figure 11b and Figure 12b). The B-factor analysis revealed no significant fluctuations, which suggests fewer loops. The eigenvalues linked to each normal mode serve as a representation of the molecule’s stiffness in motion (Figure 11c and Figure 12c). Lower eigenvalues signify simple deformation, whereas higher eigenvalues are related to higher variance. The covariance map shown in Figure 11d and Figure 12d was calculated using Cartesian coordinates Cα, and the red color denotes that the motion of the residues is correlated, while the blue and white colors are anti-correlated and uncorrelated, respectively. Individual variances are represented by the color red and cumulative variances are represented by the color green in the variance plots (Figure 11e and Figure 12e).

## 6. Discussion

In dairy cattle, the health status of the mammary gland is primarily determined, qualitatively and quantitatively, by milk production. SCC is considered an important inflammatory biomarker of bovine mammary glands. In the case of microbial invasion, a major proportion of cell subpopulation that originates in the blood by the process of chemotaxis and diapedesis are found to be the neutrophils [22,23]. In the current work, a substantial increase in milk SCC was observed in SCM dairy cows as compared to the healthy group. This change can be linked to the inflammatory response produced by the udder and is directly related to the neutrophil influx. Previous work supports the current study findings [24,25,26]. Different studies have reported that due to the inflammation in the udder, total SCC increases in milk [27,28,29]. The overall ROC data had a cut-off point < 202 with an AUC of 1, which is considered to be ideal for an model. The test also had a sensitivity and specificity of 1 (100%) which is considered excellent. The findings of ROC analysis of milk SCC in this study are in contrast to the results of previous research data [30]. The International Dairy Federation had recommended that in sub-clinically infected quarters the mean values present were 500,000 cells/mL of milk and above [31]. In South Africa, a study reported a cut-off point of 150,000 cells/mL of milk, and the ROC curve analysis in composite milk samples revealed sensitivity and specificity of 65.3% and 66.8%, respectively, with AUC of 0.7084, which indicates SCC to be a good indicator of mammary gland infections [32].

In the current work, a significant increase in the concentration of milk ferritin was found in SCM, as compared to healthy animals. The increase in ferritin concentration in SCM milk is due to the death of epithelial cells which release ferritin into the milk, thereby raising its levels [33]. Similar findings of increased milk ferritin in SCM have been reported by Orino et al. (2006), which are in agreement with the current work [34]. A similar substantial increase in the concentration of serum ferritin was found in SCM-affected animals as compared to the healthy group. Serum ferritin is considered a sensitive gauge of total body iron stores with relatively low concentrations (<1 μg/mL), whereas elevated extracellular ferritin levels are seen in inflammatory or malignant diseases [35]. Our findings are in agreement with the studies of Ornio and Watanabe (2008), who also reported high ferritin concentration in sera of SCM animals as compared to healthy animals. Thus, the determination of ferritin in sera of animals can serve as a useful indicator for the detection and prognostic forecast of intramammary infections in dairy cows [35]. Our study found that the sensitivity and specificity of ferritin in milk and sera of dairy animals were comparatively good.

In this study, a significant increase in milk CRP concentration was seen in SCM dairy cows as compared to the healthy group. Our findings are in agreement with the findings of [36]. During inflammatory conditions, minimal changes occur in the concentration of serum CRP in cattle. However, few studies have reported it as a potential marker of mastitic milk, but the availability of limited data on CRP in healthy versus mastitic milk and also its correlation with other biomarkers of mastitis, particularly SCC and other APPs of milk, hinder its use in diagnosis [37,38]. Based on the ROC curve analysis, CRP showed a sensitivity and specificity of 93% and 96% in milk, and 98% and 100% in serum, which suggests that CRP can be used as a reliable biomarker for both milk and serum for identifying SCM in dairy cows.

Further, a significant difference in concentration of Malb in milk and serum was found in mastitis-infected dairy cows when compared with the healthy animal group. The reports of previous studies suggest that Malb can be considered a biomarker of oxidative stress [39]. However, in the case of dairy cows, no studies of Malb in milk and serum were found and the same is the case with bovine SCM. More studies on Malb as an APP in dairy cattle are required to validate its role in SCM.

Bovine mastitis is a multifactorial disease with bacteria being the most common causative agent; henceforth, antimicrobials are routinely given to infected cows with mastitis (clinical or subclinical) for the treatment process. A great hurdle in the control of mastitis is the development of resistant bacteria mostly due to the non-judicious use of antibiotics, which poses a great threat to humans. In such a scenario, natural products are the only alternative to these drugs as they are structurally optimized by nature to perform specific functions. The potential of computational methods as versatile tools in the development and discovery of drugs has been recognized and known for decades.

In the drug discovery process, ADMET studies are particularly significant [40,41]. A compound must follow Lipinski’s rule of five parameters before it can be developed as a drug moiety and if a substance fails in more than two parameters, it cannot be developed as a drug [42]. A good drug molecule should be absorbed when necessary and distributed evenly throughout the body for effective metabolism and action. Drugs that fail in clinical trials do so because of toxicologically-induced adverse properties. Since they are linked to intestinal permeability and dissolution, bioactive molecules’ drug-like characteristics are especially crucial [43].

In the present study, Chaetoglobosin U was the most effective compound that showed the highest binding affinity against bovine ferritin and albumin. Various research studies have reported a wide array of biological properties for chaetoglobosins that include anti-inflammatory, antibacterial, antifungal, phytotoxic, antitumor, anti-HIV, and nematicidal [44,45,46,47,48,49,50].

To estimate ligand-binding sites in the current study, CASTp identified voids and pockets on the surface of proteins [51]. The functional regions and surface characteristics of proteins can be studied using this tool. Several research studies on immune disorders, cancer therapeutics, and studying signaling receptors (figuring out how drugs work, analyzing protein interactions, and developing computational tools) have been conducted [52,53,54,55,56]. In the current study, the protein structure’s stability and motion were assessed using the iMODS tool. The normal mode analysis provides a simple explanation for the dynamic simulations of the macromolecular complexes. The lower eigenvalues show how the molecular motion in the binding side of the interaction is stable and adaptable. It is evident from the molecular dynamic analysis that the structure exhibited a good deal of deformability.

## 7. Conclusions

In conclusion, CMT, electrical conductivity, pH, and SCC of milk can be reliable indicators of SCM. APPs (ferritin, CRP) in milk and serum have the potential to be used as markers of mastitis. However, more studies on Malb as a marker need to be conducted to validate its role in SCM. The estimation of APPs can prove a rapid and sensitive diagnostic tool to identify SCM and monitor herd health. ROC curve analysis depicted that SCC and acute phase proteins in milk and serum have strong predictive properties and could be used to develop a biomarker model for identifying SCM-infected animals from healthy animals. The drug-like and physiochemical properties of the bioactives were revealed by ADMET analysis. Molecular docking revealed Chaetoglobosin U was the most effective compound. The topographic properties of the proteins were determined by the CASTp server. The molecular dynamic approach depicted the dynamic behavior of the protein molecules. The present study demonstrated that Chaetoglobosin U could serve as an alternate molecule and can be repurposed to treat inflammatory diseases. However, further studies are required to validate its role in combating inflammatory states.

## Figures and Tables

**Table 1:** Overall distribution of CMS and volume required to perform various analysis.

S. No	Tests	Volume
1	Screening tests/cow-side tests (CMT, EC, and pH)	10 mL
2	Somatic cell count (SCC)	2 mL
3	Acute phase proteins analysis	3 mL
Total		15 mL

**Table 2:** Milk somatic cell count as a gold standard test to detect SCM.

Test	SCC Range	Samples Tested (*n* = 135)	
		Healthy	SCM
SCC (cells/mL)	When the milk SCC was <200,000 cells/mL, the animals were considered healthy. Similarly SCC > 200,000 cells/mL, the animals were considered as SCM.	25	110

**Table 3:** Descriptive statistics of milk SCC in healthy and subclinical mastitis dairy cows.

	Animal Health Status	Mean	SEM	Median	SD	Min	Max	Skewness
Somatic cell count SCC cells/µL	Healthy	180.2 ^b^	4.12	184	20.60	114	200	−1.81
	Subclinical	348.3 ^a^	17.27	300	181.1	203	1473	4.04

Means in the same columns with different superscripts are significant (*p* < 0.001); SEM: standard error of the mean; SD: standard deviation; Max: maximum; Min: minimum, SCC: somatic cell count.

**Table 4:** Descriptive statistics of milk acute phase proteins in healthy and subclinical mastitis dairy cows.

APP	Animal Health Status	Mean	SEM	Median	SD	Min	Max	Skewness
Ferritin (ng/mL)	Healthy	5.07 ^b^	0.19	5.01	0.98	3.26	6.96	0.66
	Subclinical	35.10 ^a^	1.89	29.69	19.77	10.51	90.37	1.28
CRP (pg/mL)	Healthy	4.64 ^b^	0.34	4.18	1.71	1.33	10.00	1.44
	Subclinical	20.19 ^a^	1.14	17.50	11.97	6.31	64.52	1.59
Malb (pg/mL)	Healthy	1.59 ^b^	0.06	1.68	0.33	0.94	2.00	−0.75
	Subclinical	5.03 ^a^	0.21	4.44	2.21	1.65	10.75	0.96

Means in the same columns with different superscripts are significant (*p* < 0.001); SEM: standard error of mean; SD: standard deviation; Max: maximum; Min: minimum, APP: acute phase proteins (ferritin; CRP: C-reactive protein; Malb: microalbumin).

**Table 5:** Descriptive statistics of acute phase proteins in serum of healthy and subclinical mastitis dairy cows.

APP	Animal Health Status	Mean	SEM	Median	SD	Min	Max	Skewness
Ferritin (ng/mL)	Healthy	5.67 ^b^	0.28	5.26	1.44	4.26	9.56	1.51
	Subclinical	14.54 ^a^	0.77	13.16	8.11	4.86	55.36	1.93
CRP (pg/mL)	Healthy	5.42 ^b^	0.47	5.04	2.39	2.57	10.14	0.74
	Subclinical	29.71 ^a^	1.25	24.83	13.21	9.71	68.46	0.63
Malb (pg/mL)	Healthy	1.77 ^b^	0.08	1.73	0.33	1.01	2.51	−0.15
	Subclinical	5.61 ^a^	0.21	5.19	2.20	1.66	11.85	0.90

Means in the same columns with different superscripts are significant (*p* < 0.001); SEM: standard error of mean; SD: standard deviation; Max: maximum; Min: minimum; APP: acute phase proteins (ferritin, CRP: C-reactive protein, Malb: microalbumin).

**Table 6:** ROC table of milk SCC and acute phase proteins (milk and serum).

Parameter	Cutoff	Sensitivity	Specificity	Area under Curve (AUC)	*p* Value
Milk SCC	202	100% (1.00)	100% (1.00)	1.00	<0.001
Acute phase proteins (milk)					
Ferritin	8.74	100% (1.00)	100% (1.00)	1.00	<0.001
C-reactive Protein (CRP)	8.82	93% (0.93)	96% (0.96)	0.99	<0.001
Microalbumin (Malb)	2.03	96% (0.96)	100% (1.00)	0.98	<0.001
Acute phase proteins (serum)					
Ferritin	6.79	92% (0.92)	84% (0.84)	0.95	<0.001
C-reactive Protein (CRP)	10.98	98% (0.98)	100% (1.00)	0.99	<0.001
Microalbumin (Malb)	2.63	95% (0.95)	100% (1.00)	0.98	<0.001

**Table 7:** Correlation coefficients between SCC and APP’s in milk (Ferritin, CRP, Malb) in healthy and SCM dairy cows.

Parameters	SCC	Ferritin	CRP	Malb	*p* Value
SCC	1	0.26 **	0.20 *	0.22 *	0.000
Ferritin	0.26 **	1	0.31	0.35	0.002
CRP	0.20 *	0.31	1	0.23	0.022
Malb	0.22 *	0.35	0.23	1	0.011

** Correlation is significant at the 0.01 level (2-tailed). * Correlation is significant at the 0.05 level (2-tailed). SCC: somatic cell count; CRP: C-reactive protein; Malb: microalbumin.

**Table 8:** Correlation coefficients between milk SCC and serum APP’s (Ferritin, CRP, and Malb) in healthy and SCM dairy cows.

Parameters	SCC	Ferritin	CRP	MAlb	*p* Value
SCC	1	0.28 **	0.17	0.17	0.000
Ferritin	0.28 **	1	0.18	0.22	0.001
CRP	0.17	0.18	1	0.31	0.051
Malb	0.17	0.22	0.31	1	0.054

** Correlation is significant at the 0.01 level (2-tailed). SCC: somatic cell count; CRP: C-reactive protein; Malb: microalbumin.

**Table 9:** Drug-like properties of bioactive compounds based on Lipinski’s rule of five.

Compound	Molecular Weight (g/mol)	H-Bond Donors	H-Bond Acceptors	*A log P*	TPSA Å^2^
Asperflavin	288.29	3	5	2.13	120.95
Asperlin	212.20	0	5	0.18	86.98
Austinolide	434.44	1	9	0.83	178.89
Cordyol E	244.29	1	3	3.80	106.60
Khusinol B	238.37	2	2	2.60	104.73
Luteoride E	300.35	2	4	3.22	128.83
Cytochalasin E	495.57	2	7	3.08	115
Chaetoglobosin U	528.64	3	5	4.43	112
Penicillin G	334.39	2	4	0.86	137.78
Doxycycline	480.90	6	9	−0.08	194.58

**Table 10:** ADMET analysis of bioactives and drugs.

	HIA	BBB	Water Solubility	CYP2D6	Hepatotoxicity	Ames Toxicity	Caco2	*log* KP
Asperflavin	91.03	−0.74	−3.17	No	No	No	1.1	−6.43
Asperlin	100	−0.03	−1.27	No	No	Yes	0.89	−2.99
Austinolide	88.27	−1.07	−4.37	No	No	No	0.58	−2.96
Cordyol E	92.67	−0.10	−3.87	No	No	Yes	1.89	−2.51
Khusinol B	94.78	−0.05	−3.50	No	No	Yes	1.69	−2.77
Luteoride E	91.63	−0.33	−3.75	No	Yes	Yes	0.96	−2.83
Cytochalasin E	100	−0.56	−4.93	No	No	Yes	0.84	−3.18
Chaetoglobosin U	93.98	−0.48	−4.54	No	Yes	No	1.06	−2.79
Penicillin G	59.90	−0.86	−2.47	No	Yes	No	0.11	−2.73
Doxycycline	44.27	−0.93	−2.50	No	No	No	0.14	−2.73

**Table 11:** Binding affinity of fungal bioactives against bovine ferritin and albumin.

Compounds	Binding Affinity (kcal/mol)	
	Ferritin	Albumin
Asperflavin	−8.0	−6.8
Asperlin	−6.3	−5.4
Austinolide	−7.4	−6.7
Cordyol E	−7.4	−6.2
Khusinol B	−7.3	−5.7
Luteoride E	−8.5	−5.7
Cytochalasin E	−9.3	−7.5
Chaetoglobosin U	−10.1	−8.5
Doxycycline	−8.1	−6.9
Penicillin G	−7.9	−6.4

**Table 12:** CASTp data statistics of bovine ferritin and albumin.

**Ferritin**				
**Poc ID**	**MS Volume**	**Pocket MS Area**	**Openings**	**Mouth MS Area**
1	183.5	127.0	1	76.2
2	220.4	184.2	3	83.5
3	253.6	268.8	1	21.6
4	253.7	268.9	1	21.6
5	233.4	230.2	2	54.6
**Albumin**				
**Poc ID**	**MS Volume**	**Pocket MS Area**	**Openings**	**Mouth MS Area**
1	26,186.5	10,694.3	13	3035.5
2	3061.5	2196.2	3	359.5
3	1292.6	1077.3	3	151.9
4	572.0	371.7	2	102.6
5	836.2	681.0	6	211.2

## Data Availability

All the data generated has been published in this article.

## Abbreviations

APP: acute phase proteins; APR: acute phase response; AUC: area under curve; CMT: California mastitis test; BBB: blood–brain barrier; CMS: composite milk sample; CRP: C-reactive protein; CV: coefficient of variation; DCC: Delaval cell counter; ELISA: enzyme-linked immunosorbent assay; HIA: human intestinal absorption; ROC: receiver operating characteristics; RPM: revolutions per minute; SCC: somatic cell count; SCM: subclinical mastitis.
